# Energy efficiency of engines and appliances for transport on land, water, and in air

**DOI:** 10.1007/s13280-015-0734-9

**Published:** 2015-12-14

**Authors:** Samuele Furfari

**Affiliations:** Polytechnic School, Université Libre de Bruxelles, Avenue Franklin Roosevelt 50, 1050 Brussels, Belgium

**Keywords:** Aviation, Energy for transport, Energy efficiency, Transport scenarios, Fuel for transport, Passenger transport, Road transport

## Abstract

The transport sector is fundamental for the economy but also for personal life. With a growing population and the globalization process, it is not surprising that the demand of transport is set to grow in the near future and certainly until 2050. This paper focuses on the huge potential of progress in the sector of technology for transport. As the principal sector for transport will remain on roads, the paper emphasizes the progress in the automotive sector. Since car manufacturers are investing massively into research and technology development to offer ever more efficient cars—not only energy efficient but also efficient in terms of safety and comfort—the car of tomorrow will be very different from the present one. The increasing role of electronics in cars will synergistically cooperate with that of so-called smart cities. The potential development of methane in the transport sector, mainly used for heavy transportation is discussed.

## Introduction

Mobility is fundamental to human activities in the modern society. In a globalized and interconnected world, the need for mobility will only increase. The objective now is to enhance mobility while at the same time reducing energy consumption. Oil products represent about 96 % of all energy consumption in the transport sector. It is therefore evident that this is the sector to focus on for a different future. EU’s oil import bill—which is up to 1000 million euro per day in 2014—leads to a significant deficit in the EU trade balance of around 2.5 % of GDP. Promoting energy efficiency in oil product use and a shift toward others fuels are two avenues worth considering.

Too often in Europe, the emphasis of transport issues has been placed on urban transport, while the main challenge will probably be the need of transportation of goods. Accordingly we will have to consider any innovations happening in this subsector of transport. In the transport sector, technical solutions usually do not appear in the form of a technology revolution emerging from fundamental research. Progress will very likely be based on a sequence of smaller steps rather than radical innovations. Furthermore, since the transport sector is part of a globalized industry, solutions can only be massively implemented if they are accepted globally.

## Prospects of transport demand

**Fig. 1:**
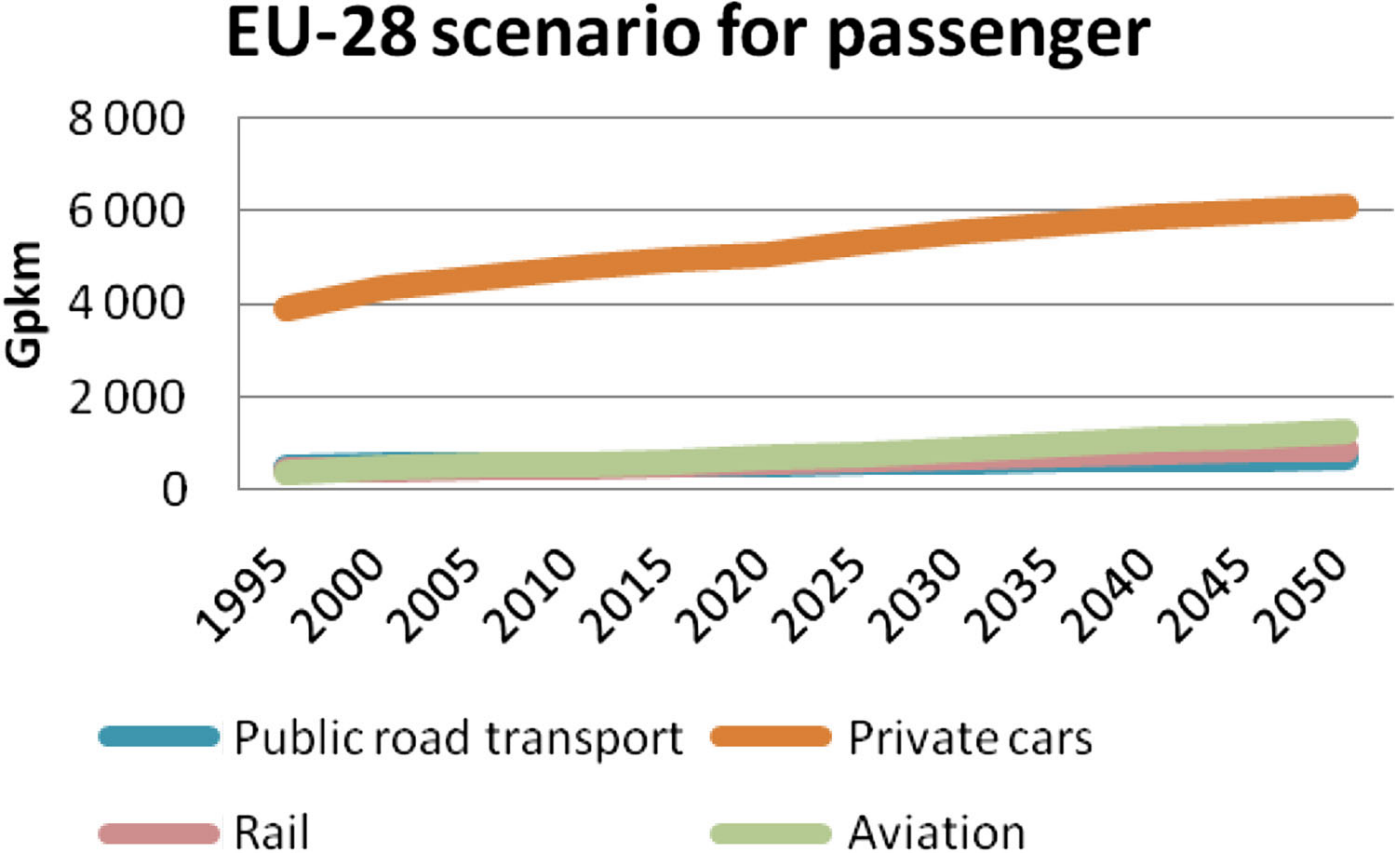
EU scenario for passenger transport from 1995 to 2050 in units of giga-passengers per km for public road transport, private cars, rail, and aviation (from STTP 2012)

**Fig. 2:**
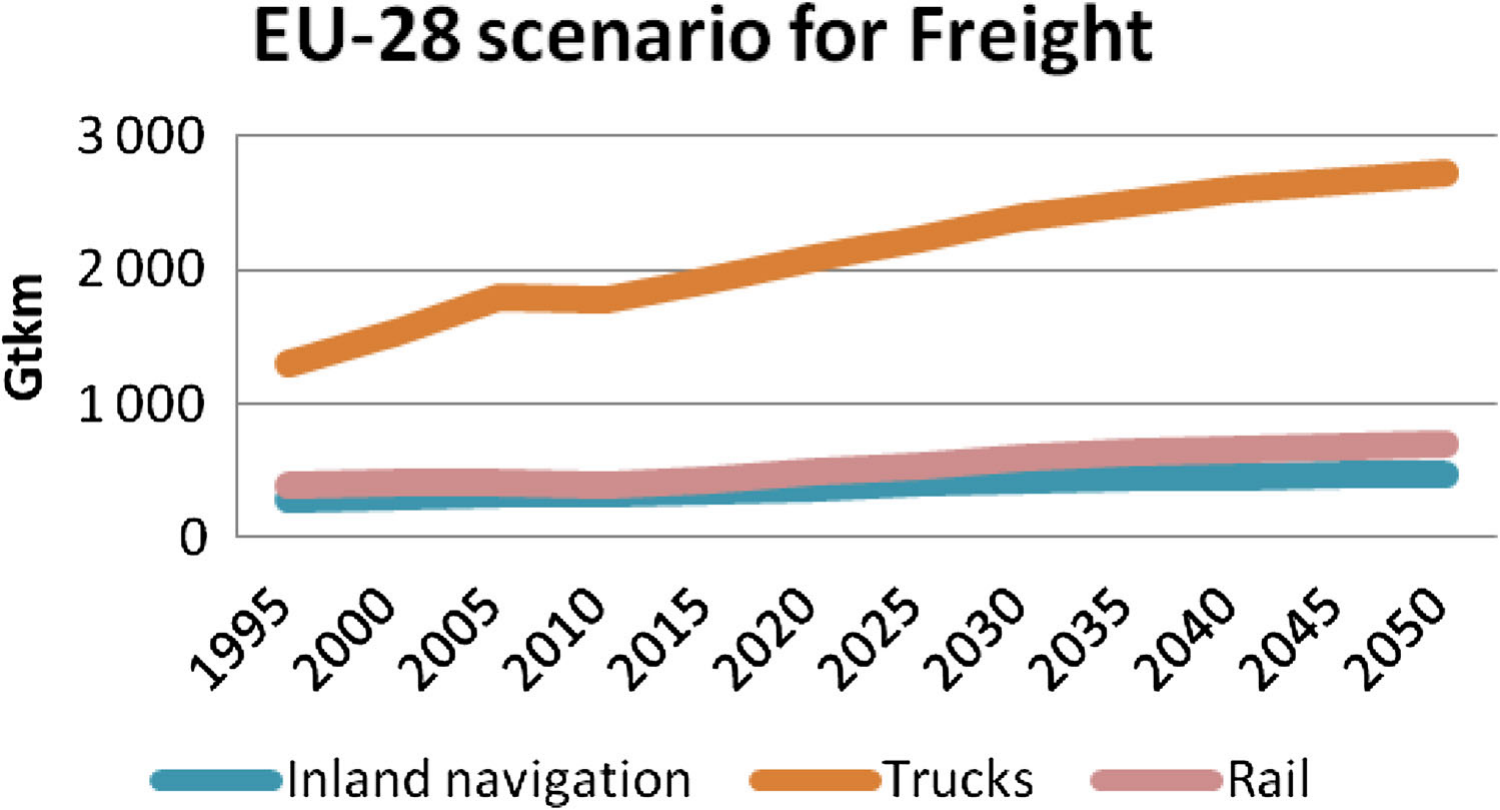
EU scenario for freight transport from 1995 to 2050 in units of giga-ton per km (from European STTP 2012)

According to European Commission scenarios, the forecasts for transport indicate a steady growth for private cars (Fig. 1) (European STTP 2012). The progress is similar for public transportation. An even stronger growth is expected for the aviation sector. In 2050, the passengers-km (i.e., the distance traveled by passengers on transit vehicles determined by multiplying the number of unlinked passenger trips by the average length of their trips) transported by private cars is expected to be 9.1 times more than the one by public road transportation and 6.9 times more than train transportation (Fig. 1). On the freight side, the increase is also steady for trucks. In 2050, transport by trucks will be 3.9 times more than by rail (Fig. 2). Others scenarios presented by IEA (2014) and BP (2015) and the US EIA (2013) are also presenting similar outlooks. This indicates that the sector of road transport deserves all our attention when we deal with energy and transport. Consequently, this article will mainly address road transport for both passengers and freight.

### Methodologies to define efficient transportation

Usually the well-to-wheels (WTW) calculation methodologies pursue the objectives of estimating the energy use and the industrial costs of all options for automotive fuels and power-trains. This complex analysis is intended to serve as a sound and broadly accepted scientific reference for research into and policy for transportation. The WTW differs from the life cycle analysis (LCA) approach, as it does not consider energy and emissions involved in building facilities and the vehicles themselves, neither end of life aspects. Therefore, the WTW focuses on fuel production (Well-to-Tank—WTT) and the vehicle use (Tank-to-Wheel—TTW) that are the major contributors to lifetime energy use. There are presently different methodologies that deliver a range of results. It will be appropriate to try to harmonize them in order to provide a technology and policy-neutral methodology leading to increased confidence in the results. Owing to the complexity of this analysis and despite of years of work in this field with detailed and accurate results, the WTW process still represents an approximation of reality (Edwards 2013).

## Road transport

### Improvement of vehicle concepts

According to the European Commission the sector investing most money into research and development is the automotive industry,^1^ a sector ranked first in the EU and third worldwide. Among EU’s top 10 R&D investing companies in 2011, four are car manufactures and part of the supply industry (all German). Among the 10 largest companies in the world, we can find one Japanese, one European and one US automotive producer.

In terms of road transport, technology improvements of the internal combustion engine and aerodynamics of the vehicle can lead to efficiency gains. If improvements in car and trucks follow the trends of the last decades, we can await a significant reduction of energy consumption. By 2050, it is possible to expect 30 % reduction in fuel consumption thanks to improvements of the internal combustion engine. Progress is underway to conceive engines running with two cylinders while other research is addressing the possibility to electronically deactivate some cylinders in a multi-cylinder engine. Electronics has become a centerpiece of automotive technology (it represents 35 % of a car’s cost in Europe) and will play a growing role in operating the engine at even lower consumption levels. A better control of injection and ignition is expected to deliver substantial energy consumption. The use of electronics not only for the engine but also all over the vehicle with sensors, controllers, and other new innovations will further improve existing systems.

A further path for progress is making the vehicles lighter using new materials thanks to the development of composite materials and carbon fiber technology. New high strength steels achieve weight savings of 30 % compared to conventional cold forming grades without reducing the safety performance. Therefore, fundamental research into materials is a key factor in transportation. One particularly effective way to increase energy efficiency of automotive vehicles is reducing their dynamic performance. It is also crucial to reduce energy consumption from heavy-duty transportation in the EU. Research into advanced active and passive aerodynamic properties for trucks and semitrailers is under way.

The connected vehicle of the future will interact via Intelligent Traffic Infrastructure with other cars to circumnavigate traffic jams and will thus contribute to limit fuel consumption. An additional aim of the connected vehicle is to improve safety. To integrate cars into “cloud-computing” and the development of eco-driving support systems will also allow to find and pay parking places or fuel station or to pay for congestion-based roads. It is estimated that 10 % improvement of fuel economy will thus be possible. Fuel consumption of vehicles also depends on tires. Research has already given results and a R&D program is still ongoing, particularly in using new types of carbon blacks that ensure a good adherence to the road while limiting the energy consumption.

### Improvement of efficiency of ancillaries

Relevant improvements during recent years have been made in the transmission systems, including automatic gear boxes, but innovative engineering is ongoing and will require more onboard electricity generation. The generation of electricity in a car engine has a very low efficiency as a result of the complexity of the process: the chemical energy of the fuel is transformed into heat, then into mechanical energy first in the engine and then in the alternator to finally generate the electricity. This is a relevant field of research but challenging because normally only inexpensive solutions are applied in the automotive industry (probably delaying the use of fuel cells). A drastic improvement will be to directly convert the heat energy of the exhaust gas into electricity using the thermoelectric effect.

Energy consumption of lighting (like LED) and other auxiliary equipment may be considered of only incremental improvement but these steps are nevertheless very important as they affect numerous applications. Design engineers are working on all these topics with the aim to reach a reduction of up to 10 % of the total fuel consumption of a vehicle through improvements of its auxiliary equipment.

### Oil-based fuels

The balance of gasoline and diesel engines has a strong impact on oil refineries. Oil refineries in Europe are challenged by the development in other parts of the world with more modern installations and less stringent rules. Already today due to the competition between diesel and gasoline, EU needs to import diesel oil. Accordingly, finding solutions for rebalancing the diesel-gasoline share is also demanded, e.g., by improving diesel engine efficiency when running on gasoline. Liquefied petroleum gas, LPG, a by-product of oil production and oil refining, is a clean transport fuel that needs to be kept and if possible expanded.

### Compressed natural gas (CNG) and liquefied natural gas (LNG)

Due to their higher energy density, oil products have been widely and globally preferred. In 2013, 17.7 million vehicles in the world used natural gas, the vast majority of which (16.3 million) were light commercial vehicles running mostly on compressed natural gas (CNG). Iran, for example, which does not have the refining capacity to produce motor fuel and cannot export its gas, has decided to use natural gas in the transportation sector: it has 3.3 million vehicles running on natural gas, or 27 % of its total fleet. In addition to Iran, six other countries have more than 1 million gas-powered vehicles: Pakistan (2.8 million), Argentina (2.2 million), Brazil (1.7 million), China (1.6 million), and India (1.5 million). While the development of CNG started to emerge in Europe, the development of shale gas in USA has generated a strong interest for liquefied natural gas (LNG) for transportation. Shale gas producers realized that their truck fleets can operate with the fuel that they are actually producing. This has prompted a strong development in the use of LNG also in others fleet sectors (e.g., parcel deliveries and school buses). In 2013, the European Commission launched the LNG Blue Corridors project,^2^ which will run until 2017 and which aims to establish the LNG as a real alternative for medium- and long-distance transport—first as a complementary fuel and later as an adequate substitute for diesel. The program provides for the construction of a fleet of around 100 LNG heavy-duty vehicles meeting EURO IV standards^3^ along with 14 new stations along four corridors with an LNG refueling station every 400 km on average.

This is now reality also for trains and ships. There are already 7 million of operating hours of ships with LNG engines, still restricted mainly to small ships. There are also 210 LNG installations in ports worldwide. This is beneficial both for reducing emissions (CO_2_, NOx, but especially 100 % of SO_2_, and 100 % of particulate compared with diesel engines) and improving or maintaining competitiveness. Engine manufacturers are working in developing more efficient small-scale and large-scale LNG engines for ships. The awareness of port authorities for this clean transport fuel is rising also because the EU has decided (Directive 2014/94/UE, 2014) that by 2025 all Trans European Network core ports will have to provide LNG as fuel. Indeed there are a lot of synergies between ports, trains, and trucks in the field of LNG transportation; they need to be identified also to justify further development of LNG infrastructures for maritime purposes. For long haulage, it might take longer before LNG is widely used (except for the ongoing LNG ship carriers that are already operated with naturally evaporated gas).

### Electricity

Typically, conventional gasoline engines effectively use only 15 % of the fuel energy content to move the vehicle or to power accessories, and diesel engines can reach onboard efficiencies of 20 %, while electric vehicles have an onboard efficiency of around 80 %. Bear in mind that from 1890 to 1910, the electrical car surpassed any other type of vehicle. After the end of the first World War, battery-operated vehicles disappeared. Thereafter, a number of tries with electrical cars could be observed but batteries remained unreliable to use in the car industry. To overcome these drawbacks, current research focuses on the development of new solid electrolytes (polymer) and electrodes that are resistant to passivation of the batteries. The unique characteristics of graphene, including its excellent electrical conductivity and high surface area, could make it an ideal medium for the electrodes of batteries. A breakthrough in this technology can be expected if low capacity losses after multiple charge and discharge cycles, and a long-life can be demonstrated. Of course, mass production of such batteries has to yield a compatible price.

#### Supercapacitors

While the batteries are electrochemical, capacitor devices are electrostatic. Another advantage is that they can handle up to one million charge- and discharge cycles without degeneration. Charging can be done in less than a second. The disadvantage is that the amount of energy is limited, and the time the charge can be stored is only minutes. A version of capacitors, which eliminates some of these drawbacks, is so-called supercapacitors. In order to overcome the limited availability of lithium, research is now focusing also on sodium. In addition to the lifetime limitation of present batteries, their too low output voltage is a disadvantage. In the future, organic chemistry and electrochemistry will play a fundamental role in the production of energy and electricity in particular. Therefore, these disciplines of the chemistry need to receive proper attention.

#### Range extender

In a hybrid car, the torque on the wheel is given by an electric engine and an internal combustion engine working in tandem. In the Range Extender, it is always the electrical engine that drives the wheels, where the energy to run the electrical engines always coming from a battery. When the charge of the battery is too low (usually 30 %) then an internal combustion engine drives an alternator to charge it up again. As they are smaller and simpler than the internal combustion engines used in conventional vehicles, Range Extender are considered at present to have advantages.

### Fuel cells and hydrogen

Fuel cells will be a better option than the internal combustion engine from the day when price and reliability are acceptable. Fuel cells are a well-known technology in steady-state electricity production. However, they have are not yet been accepted widely as an economic solution. Despite years of efforts they are still unreliable and expensive. Reducing production costs remains the main challenge because their production is difficult and cannot be automated. Furthermore, improving their lifetime is also a problem. Once steady-state fuel cells will be competitive for electricity generation, then the devices have to be miniaturized for the use in cars.

Hydrogen vehicles are hardly a solution with current technologies. As hydrogen needs to be produced from hydrocarbons, or with electricity, the process of gas reforming or water electrolysis cannot be considered as a sustainable solution at present due to the accumulation of efficiency losses. The line of research is therefore to transform directly the energy of the photon into chemical energy by its action on water. Research efforts on artificial photosynthesis (photochemical catalysis) are also ongoing but it appears to be a feasible solution only in the long term. Research on the photo-electrochemical catalysis is promising in this regard. It is hoped that through a catalyst, which accelerate slow processes, liquid fuels can directly be produced from solar energy. With sun and water, it should be possible within a few decades to produce artificial fuels even in poor countries. Longer-term research is also explored with gas reforming coupled with CCS. The principles are known but the miniaturization and mass production have still to be demonstrated.

## Water transport

### Improvement of efficiency

The improvement of energy efficiency in ships does not concentrate exclusively on the efficiency of the large engine but considers design and operation of the whole boat as well as the use of lost heat (co-generation). There is a need to develop standardized infrastructure worldwide, and it would be wise to have an international alliance to develop common standards.

### Alternative fuels

The huge potential in the use of LNG has already been mentioned. This is also a field of research in the military sector. In countries, where a lot of biomass is available (like in Scandinavia), research is ongoing to produce methanol produced from forestry products with the aim mainly to fuel ships. More generally, research to produce second-generation biofuels is ongoing to exploit 100 % of the vegetal material and not to limit the conversion of the more profitable part of it. It is only if we succeed in converting all the biomass into a set of derived fuels that economic and sustainable success can be reached.

## Air transport

Although the current share of aviation fuel demand in total world oil demand is only about 5.8 %, its growth rate causes 2.7 million barrels per day incremental oil demand by 2030 (Mazraati 2011), despite impressive energy efficiency improvements of 3 % per year. In the aircraft industry, aluminum is continuously substituted by composite materials. However, the aircraft structure has to withstand the electromagnetic environment it is exposed to. A series of research projects to develop techniques, which are intended to partially recover some of the beneficial properties of metallic structures, without too much weight and cost penalty are underway. Multifunctional materials and nanotechnology are considered key development areas for the use of composite materials in commercial aviation. The use of carbon nanotubes for improvement of electrical conductivity needs to be further pursued. Similarly, graphene technology shows a high potential for composite airframe improvements.

Improving the efficiency and performance of gas turbines is a very expensive effort needing long development periods. Improvements will require higher operating temperatures; therefore, research is focusing on materials resisting these severe conditions (an example is Tomo-Lithographic Molding manufacturing technology to produce improved airfoils). Fundamental material sciences should receive due attention.

Airplanes need a lot of electricity during operation. Onboard electricity generation is presently done by burning kerosene. Civil aviation airplane manufacturers are developing various solutions to generate onboard electricity more efficiently. Onboard H_2_ production from jet-fuel to generate electricity is a possible way. By catalytic partial dehydrogenation of the fuel, hydrogen can be produced (Liew 2011) and then electricity can be generated with a higher efficiency thanks to fuel cell technology. The by-product of the dehydrogenation is still a very good fuel to be burned in the turbine.

## Rail transport

For the rail transport, the pollution due to the diesel trains is the major issue to tackle. On the energy side, it is also very relevant with the full electrification as the efficiency of the whole system will improve. As previously said, LNG powered trains are a promising solution for nonelectric tracks particularly for freight transportation. Improvement of electrical trains is a challenge as the technology is already well developed and very efficient. However, research is ongoing in the traction/braking systems by developing new semiconductors that will reduce dissipative losses (in conduction and commutation). Another field that will deliver efficiency improvements is the regenerative braking where the train engine operates in generator mode and returns DC electricity to the grid. The electricity generated in this way can be used by others trains circulating on the same line. Research is also ongoing to avoid to be limited by this last restriction so as to recover the energy of braking at every occasion. These efforts are all applied sciences rather than fundamental research, and engineering by industry is needed for achieving an efficient energy system for railway transportation.

## Summary

Transportation and energy are strongly linked. Without energy, there will be no modern transport system; the reverse is, however, also true. As the demand of transport will increase, energy consumption will certainly grow for a number of decades to come. Energy efficiency measures will not be able to absorb all the increase of the demand resulting from this growth. Nevertheless, progress in improving energy efficiency in the field of transport is fundamental. The need for improvement is particularly crucial for road transport because it is the most relevant sector and it will continue to expand. Car and supplier industries are investing a lot in RTD not only to increase the energy performance of their products for marketing reasons but also to cope with increasingly more stringent legislation and regulations. Electronics and interconnectivity will play a foremost role in this pursuit for efficiency. While the limit of plug-in cars is linked to the battery, hybrid cars and on-road electricity generation seem to be more attractive solutions for the car industry. Energy efficiency in all transport chains need also to be better defined; public authorities should cooperate with stakeholders to set unequivocal standards. Regarding alternative fuels, the environmental limitations of existing biofuels require more innovative solutions. It is hoped that research will accelerate the development of next generation biofuels. In the meantime, LNG appears as a readily available and cleaner alternative to oil products, especially for freight transport. Maritime and rail transport might take advantage of this abundant, affordable, and clean fuel. Energy efficiency in air transport is also a priority for aircraft manufacturers and aircraft engine producers. Innovative concepts for passenger traffic already exist—now it is time to provide a cost-effective alternative to fossil fuels for transport of goods on the roads.

## Footnotes

1 http://europa.eu/rapid/press-release_IP-11-1205_en.htm

2 (http://lngbc.eu/).

3 (https://www.dieselnet.com/standards/eu/hd.php).
